# Evaluation of Siderophores Generated by *Pseudomonas* Bacteria and Their Possible Application as Fe Biofertilizers

**DOI:** 10.3390/plants12234054

**Published:** 2023-12-02

**Authors:** José María Lozano-González, Silvia Valverde, Mónica Montoya, Marta Martín, Rafael Rivilla, Juan J. Lucena, Sandra López-Rayo

**Affiliations:** 1 Department of Agricultural Chemistry and Food Science, Universidad Autónoma de Madrid, Av. Francisco Tomás y Valiente 7, 28049 Madrid, Spain; josem.lozano@uam.es (J.M.L.-G.); silvia.valverde@uva.es (S.V.); juanjose.lucena@uam.es (J.J.L.); 2 Department of Biology, Universidad Autónoma de Madrid, c/Darwin, 2, 28049 Madrid, Spain; monica.montoya@inv.uam.es (M.M.); m.martin@uam.es (M.M.); rafael.rivilla@uam.es (R.R.); 3 Departamento de Química y Tecnología de Alimentos, Escuela Técnica Superior de Ingeniería Agronómica, Alimentaria y de Biosistemas, Universidad Politécnica de Madrid, Ciudad Universitaria, 28040 Madrid, Spain

**Keywords:** iron, siderophore, pyoverdine, *Pseudomonas*, biofertilizer

## Abstract

The application of synthetic iron chelates to overcome iron deficiency in crops is leading to a high impact on the environment, making it necessary to find more friendly fertilizers. A promising alternative is the application of biodegradable iron chelates, such as those based on siderophores. In the present work, seven bacterial strains of the genus *Pseudomonas* were selected for their ability to secrete pyoverdine, a siderophore with a high affinity for iron, which could be used as a biofertilizer. The concentration of siderophores secreted by each bacterium expressed as desferrioxamine B equivalents, and the pyoverdine concentration was determined. Their potential as Fe biofertilizers was determined based on their capacity to complex Fe, determining the maximum iron complexation capacity at alkaline pH and selecting the RMC4 strain. The biostimulant capacity of the RMC4 strain was evaluated through the secretion of organic acids such as the hormone Indol-3-acetic acid or glutamic acid, among others, in a kinetic assay. Finally, the genome of RMC4 was determined, and the strain was identified as *Pseudomonas monsensis.* The annotated genome was screened for genes and gene clusters implicated in biofertilization and plant growth promotion. Besides iron mobilization, genes related to phosphorus solubilization, production of phytohormones and biological control, among others, were observed, indicating the suitability of RMC4 as an inoculant. In conclusion, RMC4 and its siderophores are promising sources for Fe biofertilization in agriculture.

## 1. Introduction

Iron (Fe) deficiency is a major nutritional disorder in crops causing lower yields and important economic losses [1]. Despite the fact that Fe is the fourth most abundant element on Earth, Fe deficiency has been considered the most common micronutrient deficiency in crops worldwide [1]. This problem is especially relevant in alkaline and calcareous soil conditions characterized by a pH between 7.4–8.5 [2], and a high bicarbonate concentration, buffering the pH and causing the Fe to react with insoluble chemical species, thus limiting its availability for crops [3,4]. To overcome Fe deficiency in crops, the application of synthetic compounds derived from polyaminocarboxylic acids or polyaminophenylcarboxylic acids (commonly known as Fe chelates) such as ethylendiaminetetraacetic acid (EDTA), ethylendiamine-N-N’bis(o-hydroxyphenylacetic) acid (o,o-EDDHA), or N-N’bis(o-hydroxyphenyl)ethylendiamine-N-N’-diacetic acid (HBED) [5] is the most widespread solution [4]. Despite their effectiveness, their high price and environmental impact [6,7,8] have encouraged the development of new research lines to focus on finding sustainable alternative formulations to these Fe chelates.

One promising line of research in agriculture is the use of siderophore-producing bacteria [9,10,11]. Under Fe deficiency conditions, these bacteria secrete siderophores. Siderophores are molecules with low molecular weight and high affinity and selectivity for binding and complexing with Fe^3+^ [12]. This high affinity is due to the functional donor groups present in the siderophores (amino, catecholate, hydroxamate, and/or carboxylate), which are able to bind to Fe [13]. Depending on the main functional group present in the siderophore, it can be classified as a catecholate, hydroxamate, carboxylate, or mixed (if they have more than one functional group) type [14]. Currently, many different siderophores of each type are known: catecholate types such as aminochelin, azotochelin, bacillobactin, enterobactin or protochelin; hydroxamate types such as desferrioxamine B (DFOB), putrebactin or vicibactin; carboxylate types such as corynebactin, rhizoferrin or vibrioferrin; and mixed types such as aerobactin, ferribactin, pseudobactin or pyoverdine [9,13,14]. All known siderophores are compiled in a freely usable database [15].

Siderophores exhibit a well-established high-affinity binding with Fe, yet their utilization in biofertilizers remains constrained. Ferreira et al. [16] investigated the efficacy of freeze-dried products derived from siderophore bacterial cultures (*Azotobacter vinelandii* and *Bacillus subtilis*) complexed with Fe in ameliorating Fe deficiency in soybean crops. Results indicated the superior stability of *Azotobacter vinelandii* siderophores in calcareous soils, leading to significant enhancements in dry weight and leaf chlorophyll content. This underscores the potential of *Azotobacter vinelandii* siderophore–Fe complexes as environmentally friendly Fe sources for addressing Fe deficiency in calcareous soils. While the exploration of other bacterial families such as *Bacillus megaterium*, *Pantoea allii*, and *Rhizobium radiobacter* in siderophore production and Fe complexation in calcareous conditions has been studied and dismissed [17], promising attributes have been identified in bacteria from the Pseudomonas family. Therefore, the selection of bacteria with favorable traits for Fe biofertilizers in calcareous environments may hinge on identifying those producing siderophores with strong Fe affinity and high production capacity.

*Pseudomonas* are Gram-negative bacteria, with well-known plant growth-promoting (PGP) characteristics including the production of siderophores [18], the most significant of which is pyoverdine [19]; production of 1-aminocyclopropane-1-carboxylate deaminase (ACC deaminase) [20]; production of indole acetic acid (IAA) [21]; phosphate solubilization [22]; and nitrogen fixation [7], among others. Furthermore, there are several experiments in the literature testing the effect of the PGP characteristics of *Pseudomonas* on different crops. In the experiment performed by Gusain et al. [23], several bacteria from rainfed agricultural fields of the Garhwal Himalayas were tested for inorganic phosphate solubilization, production of IAA, and production of siderophore. One of the bacteria selected was identified as *Pseudomonas koreensis*, and it promoted plant growth in rice, increasing biomass and phosphorus uptake.

On the other hand, another strategy for the application of these plant growth-promoting bacteria has been found in the literature, which consists of separating the bacterial secretions from the bacteria and isolating the compounds of interest (such as siderophores) for later application. López-Rayo et al. [24] tested the efficacy of ethylenediaminedisuccinic acid ([S,S’]-EDDS) as an Fe fertilizer. [S,S’]-EDDS is a siderophore generated by the actinomycete bacterium *Amycolatpsis japonicum* [25]. López-Rayo et al. [24] observed that the Fe concentration in soybean plants grown in calcareous soil was similar for [S,S’]-EDDS/Fe and EDTA/Fe applications. Nagata et al. [26] applied pyoverdine in Fe deficient tomato plants and observed an improvement in the bioavailability of Fe in tomato plants. Nagata et al. [26] demonstrated the increase in Fe bioavailability in tomato plants due to pyoverdine; however, they used an optimum pH for Fe nutrition (5.75) and a high Fe concentration (100 µM).

This investigation aimed to identify a bacterial strain with a high capacity to produce siderophores from horticultural soils and determine its characterization as an eco-friendly alternative to synthetic ligands for Fe chelation and potential use to alleviate Fe chlorosis in crops. To achieve this objective, wild bacteria of *Pseudomonas* were isolated from soils, and the strain producing a higher concentration of siderophores was selected. The Fe chelating capacity in alkaline soil, the production of organic acids responsible for biostimulant activity in plants, and the plant growth-promoting rhizobacteria (PGPR) characteristics were evaluated in the selected strain.

## 2. Results

### 2.1. Bacterial Isolation

Different bacterial strains of the genus *Pseudomonas* were isolated from the rhizosphere of crops. Selected colonies were identified as pseudomonads by their 16S RNA gene sequence. Isolates were also tested for the lack of growth at 37 °C, and siblings were discarded by their BOX pattern. After bacterial isolation, chrome azurol sulphonate (CAS) assay was performed in Petri dishes to determine the amount of siderophore produced by each isolated bacterium. Those strains with more halo formation than the control were selected as possible biofertilizers. The results are shown in Table 1.

Those bacteria that showed more halo formation (mm) than the control bacterium were selected: RMT9 (20 mm), RKP1 (17 mm), RKP2 (20 mm), RKP3 (20 mm), RMC4 (25 mm), RMC9 (23 mm), and HFL3 (30 mm).

### 2.2. Siderophore and Pyoverdine Production

**Figure 1:**
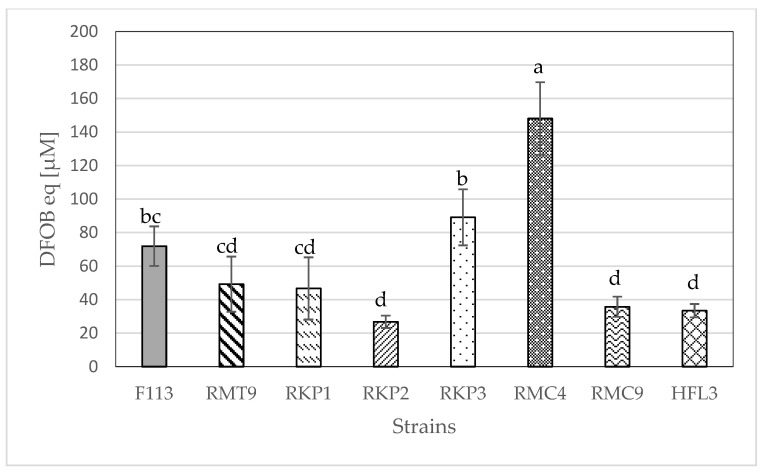
Concentration of siderophore expressed as DFOB equivalents (µM). The data are the mean ± SE (n = 9). Different letters indicate significant differences according to Duncan’s test (*p* < 0.05).

**Figure 2:**
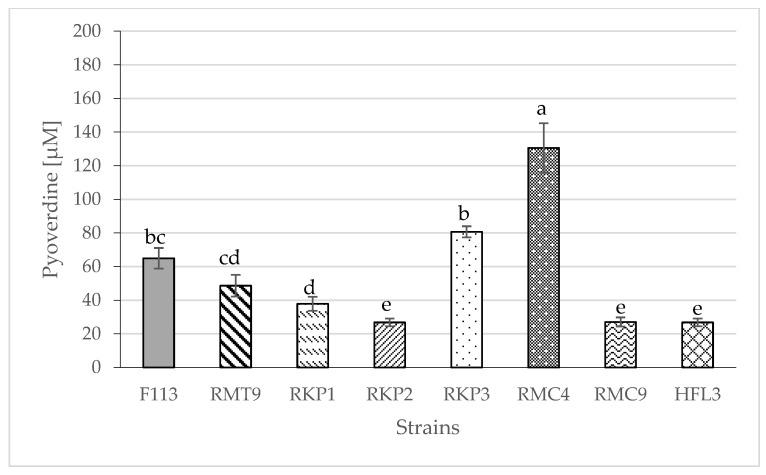
Concentration of pyoverdine (µM) produced by the selected bacterial strains. The data are the mean ± SE (n = 9). Different letters indicate significant differences according to Duncan’s test (*p* < 0.05).

With the selected bacteria described in 3.1, the CAS liquid assay was performed to quantify siderophore production; the results were expressed as DFOB equivalents (µM) and compared to the strain F113, used as a control. As can be seen in Figure 1, the strain producing the highest concentration of siderophores was RMC4. Also, this strain produced the highest concentration of pyoverdine (Figure 2). Only this strain produced a significantly higher amount of siderophores or pyoverdine than the control strain F113.

### 2.3. Iron Complexation Capacity Assays

**Figure 3:**
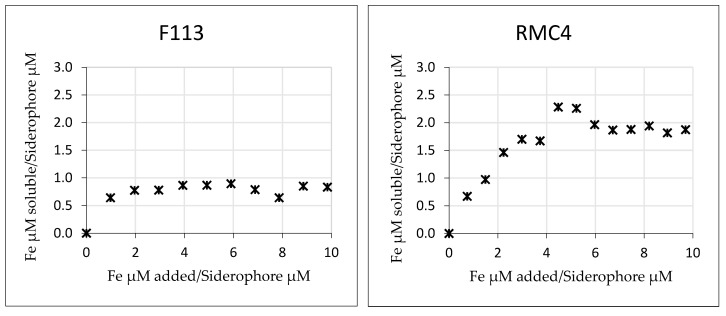
Molar ratio values of Fe: siderophore determined by the representation of the concentration of soluble Fe (µM)/siderophore concentration (µM) vs. concentration of added Fe (µM)/siderophore concentration (µM) for each bacterial strain.

To assess the potential use of the siderophores obtained from the selected strains as Fe biofertilizers, the maximum complexation capacity was determined with the spent media obtained after the growth of the selected strains. To a constant amount of bacterial extract of each bacterial strain, increasing concentrations of Fe^3+^ were added at pH 9, obtaining a curve, where the maximum Fe complexation can be determined (Figure 3). The higher the soluble Fe value, the higher the complexation capacity of the siderophore.

Once the soluble Fe was measured and the siderophore concentration of each spent medium was known, the approximated stoichiometry with which each supernatant would complex Fe was calculated, and the results are shown in Table 2. The RMC4 strain presented the highest value of stoichiometry, which indicated that, at alkaline pHs, the spent medium of the bacterial strain RMC4 could complex a maximum of 2 Fe atoms for each siderophore unit.

### 2.4. Titration of Bacterial Secretion

**Figure 4:**
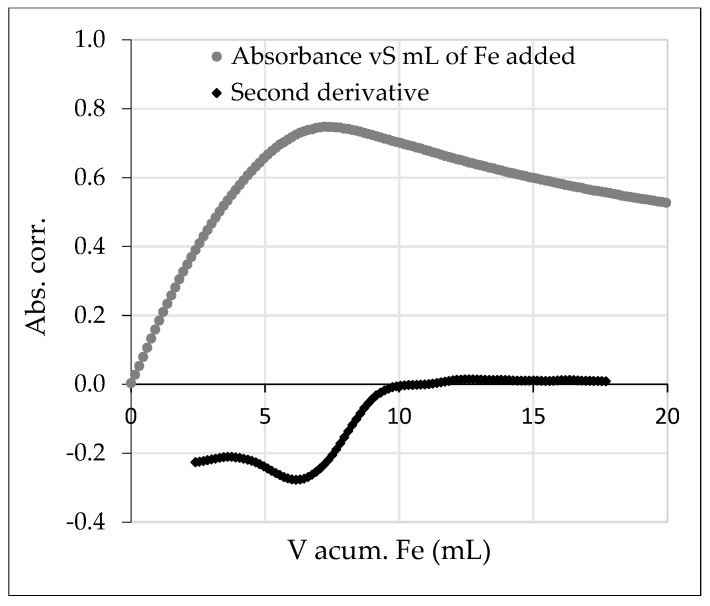
The grey circular dots represent the corrected absorbance for mL of Fe added. The rhomboid dots represent the second derivative of the absorbance versus mL of Fe added.

The RMC4 strain was selected because of its high Fe complexation capacity at pH 9. Then, a titration was performed at pH 8.0 and with a fixed wavelength at 480 nm to verify the maximum Fe complexation capacity obtained. The results are shown in Figure 4. The gradual increase in the absorbance of the solution at 480 nm indicated the formation of the pyoverdine/Fe complex attributed to the absorbance of the Fe bond to hydroxamate groups. Once the maximum complexation capacity was reached, the absorbance no longer increased; instead, a slight, gradual decrease was observed. To ascertain the value corresponding to the maximum complexation capacity, a mathematical analysis was performed by calculating the second derivative of the absorbance. The point where the minimum was achieved in this analysis represented the point of maximum complexation. According to this, the complex formed between pyoverdine and Fe had a molar stoichiometry of 1:1 (169 ± 8 µM of pyoverdine and 168 ± 5 µM of Fe).

### 2.5. Temporal Variation in Organic Acids

**Figure 5:**
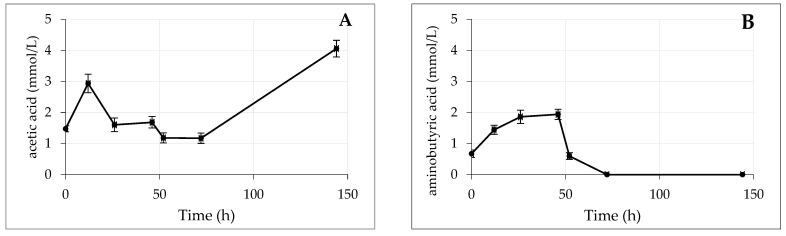

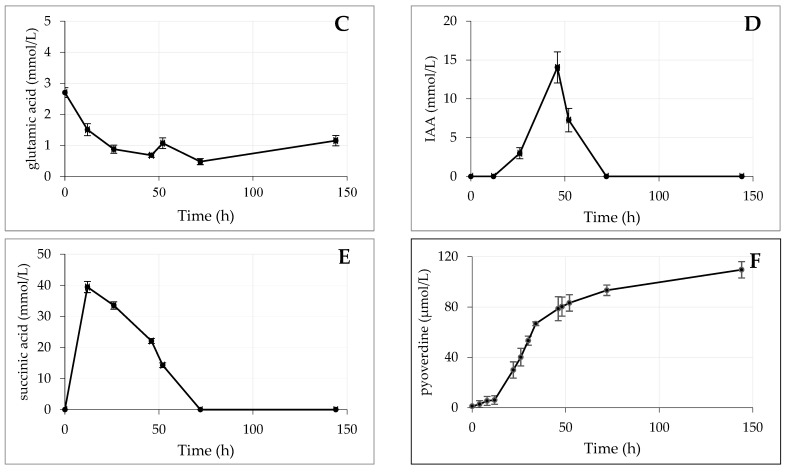
Concentration (mmol/L) of (**A**) acetic acid; (**B**) aminobutyric acid; (**C**) glutamic acid; (**D**) IAA; (**E**) succinic acid, and (**F**) pyoverdine (µmol/L) over time (n = 3) in the bacterial extract of the Pseudomonas RMC4.

Whether the RMC4 bacterial strain produces biostimulant compounds was studied thought the quantification of the organic acids produced in a kinetic assay. Results are shown in Figure 5. The analysis included a large series of acids, but only glutamic acid, acetic acid, aminobutyric acid, IAA, and succinic acid were detected in the secretions. Complementing this study, the concentration of pyoverdine was quantified by spectrophotometric methods (Section 4.2) (Figure 5F). As can be seen in Figure 5, with respect to acetic acid (5A), the concentration remained in a constant range until 72 h, where the concentration increased; aminobutyric acid (5B) showed a peak at 50 h, then its concentration decreased to 0; glutamic acid (5C) did not show marked variation in its concentration during all of the experiment; with IAA (5D), a peak concentration was obtained at 48 h, then it decreased to 0; succinic acid (5E) decreased gradually to 0 concentration at 72 h; and finally, the concentration of pyoverdine (5F) increased steadily, with different slopes up to 150 h. The presence of the IAA among others, indicated that RMC4 could have biostimulant properties.

### 2.6. Genomic Analysis of Pseudomonas monsensis RMC4

Sequencing of the RMC4 genome resulted in 16 contigs spanning 6,443,679 bp, and the sequencing coverage was estimated at 37×. A completeness of 98.4% was estimated for this genome by the SqueezeMeta pipeline. The genome was shown to belong to a strain of *Pseudomonas monsensis*, a species belonging to the *P. koreensis* subgroup of the *P. fluorescens* cluster of species.

Functional assignment of the genome showed the presence of multiple genes and clusters involved in plant growth promoting activity (Table 3). A cluster was identified in the genome for the biosynthesis of potential antifungals related to lokisin. In addition, the cluster for the hydrocyanic acid production (*hcn* genes) was also found. The potential of the strain RMC4 to produce siderophores was confirmed by the identification of the *fecAR* and *hasDEF* genes that are involved in iron siderophore biosynthesis. The genome also showed the bacteria’s ability to produce and secrete the siderophore pyoverdine, the presence of which was observed during growth of the strain under low iron availability conditions. Furthermore, genes and clusters implicated in phosphate mobilization were also found. Regarding the genes involved in phytohormone production by *P. monsensis* RMC4, the cluster composed of the *IaaM* and *IaaH* genes, responsible for the biosynthesis of the auxin IAA, was identified. Likewise, a cluster for the degradation of auxin phenylacetic acid (PAA), which may be involved role in plant–bacteria interaction, was also found. Additionally, the *fitD* gene, which encodes an insect toxin, was also detected in the genome.

## 3. Discussion

The main objective of this work was to find and characterize a bacterial strain from horticultural soils with a high capacity to produce siderophores, as an eco-friendly alternative to synthetic ligands to be used as Fe biofertilizer. Besides its Fe complexing capacity, the plant growth-promoting characteristics were also analyzed. A wide variety of naturally produced bacteria were isolated, but only those that fluoresced under UV light were selected, as this is indicative of pyoverdine-producing *Pseudomonas*. The CAS test and the comparison in halo formation were performed on the selected bacteria. The control for comparison was the bacterium *Pseudomonas fluorescens* F113, isolated for the first time from the rhizosphere of sugar beet by Shanahan et al. [27] and well-described. A variant of this bacterium, “F-variant”, was generated, which overproduced pyoverdine in an iron-limited medium (SA) and even produced pyoverdine in the iron-rich medium LB [28]. The bacteria that qualitatively generated a larger halo than the control were selected, resulting in a total of seven bacteria: RMT9, RKP1, RKP2, RKP3, RMC4, RMC9, and HFL3 (Table 1). The larger halo formation could be due to a higher production of siderophores. To verify this, a method to quantify the production of siderophores was performed (Figure 1). A colorful dye–iron complex loses its color when a compound with a higher affinity for Fe is added [29]. In an attempt to quantify this process, a known commercial siderophore, DFOB, was used. Thus, the results could be expressed in concentration as DFOB equivalents [30]. The RMC4 bacterium showed a significantly higher concentration of siderophore (DFOB equivalents) than the control bacterium F113. The rest of the bacteria did not show significant differences as compared to the control, except for RKP2, RMC9, and HFL3, which had significantly lower values than the control. Comparing the results in Table 1 and Figure 1, bacteria with a higher halo formation than F113 did not show significantly higher siderophore concentrations than the control bacterium. As one test was qualitative and the other quantitative, it would be expected that concentrations without significant differences could be obtained; however, significantly lower siderophore concentrations than that of the control were obtained. This may have been due to the difference in the culture media used: in the halo formation test, SA medium was used, while in the determination of siderophore concentration, MMS was used. MMS has been shown to cause a significant increase in pyoverdine production by *Pseudomonas fluorescens* [31]. However, this increase has not been observed in other bacteria of the *Pseudomonas* genus, such as *P. aeruginosa*, *chlororaphis*, *pertucinogena*, *putida*, *stutzeri*, and *syringae*. Sasirekha and Srividya [32] observed that the bacterium *Pseudomonas aeruginosa* FP6 produced more siderophores in a culture medium with mannitol or sucrose as a carbon source (instead of succinic acid) or with yeast extract or urea as a nitrogen source (instead of ammonium sulfate). Murugappan et al. [33] optimized the culture medium for maximum siderophore production from the bacterium *Pseudomonas putida* (CMMB2) and observed that, for this bacterium, the culture medium for maximum production of siderophores was the MM9 medium, the best carbon source was succinate (as used in this paper), and the nitrogen source was NH_4_Cl, with a pH adjusted to 8 (7 was the pH used in this work). The use of MMS may have resulted in the optimization of pyoverdine production by those bacteria belonging to the *fluorescens* species, while the maximum yield was not obtained from those not belonging to this group.

The quantification of siderophores by CAS assay may have presented interference due to compounds present in the culture medium that could also have complexed Fe (such as phosphates) and cause discoloration before the addition of DFOB. However, as the same culture medium was used for all bacteria, in combination with the application of a blank using only the culture medium, this was not likely to interfere with the results. The CAS assay is the most widely used method for the quantification of siderophores; however, due to the large number of siderophores that could be secreted by the same bacterium and the complexity of the culture supernatant, it is not possible to accurately determine the real concentration of siderophores. For the identification of each siderophore, advanced analytical techniques, such as ultraperformance liquid chromatography coupled to tandem mass spectrometry [34], would have to be used. Nevertheless, the CAS assay is a very fast, reliable, and inexpensive method of approximating the concentration of siderophores.

The RMC4 bacterium produced a significantly higher concentration of pyoverdine than the control bacterium, while RKP1, RKP2, RMC9, and HFL3 bacteria produced significantly lower concentrations of pyoverdine than F113 (Figure 2). These results are possibly related to the group of *Pseudomonas* to which each bacterium belongs, as explained above. Related to RMC4, a comparison between Figure 1 and Figure 2 showed that the percentage of siderophores corresponding to pyoverdine was 88.1 ± 8.8%; this means that the major component of the segregated siderophores in RMC4 bacteria was pyoverdine. This result is consistent with that described in the literature. Under Fe deficiency conditions, bacteria could synthesize siderophores to bind Fe^3+^ and load into the cytoplasm via highly specific transport systems [35]. If the Fe limitation conditions are moderated, bacteria could produce siderophores with low affinity for Fe but metabolically inexpensive to produce, although under extremely Fe deficient conditions, bacteria could produce highly efficient but metabolically expensive siderophores [36,37]. In the case of *Pseudomonas aeruginosa*, in situations of moderate Fe deficiency, pyochelin is secreted, an inefficient but metabolically profitable siderophore; under severe Fe deficiency, pyoverdine, a siderophore very efficient (K_LFe(III)_ = 10^30.8^; K_LHFe(III)_ = 10^43.0^) [38] but metabolically very costly, is secreted [39]. In the present experiment, severe Fe deficiency conditions were induced, causing the bacteria to tend to secrete pyoverdine rather than other siderophores with less affinity for Fe; this was consistent with the fact that a higher percentage of the siderophores excreted corresponded to pyoverdine.

A complexation capacity assay was performed, and the stoichiometry of the Fe complex was determined (Table 2). The resulting stoichiometry for RMC4 (Figure 3) was around 2, showing that the maximum Fe complexation capacity of the siderophores secreted by this bacterium is double that of F113, RKP3, and RMC9, which resulted in a 1:1 stoichiometry.

In the experiment conducted by Ferreira et al. [17] to determine the Fe complexation capacity of other bacterial strains, the method described by Villén was also used to determine the maximum Fe complexation capacity of different siderophores under alkaline conditions. Ferreira et al. [17] studied the siderophores secreted by the bacteria *Azotobacter vinelandii*, *Bacillus megaterium*, *Bacillus subtilis*, *Pantoaea allii*, and *Rhizobium radiobacter* and the maximum Fe complexation capacity by the abovementioned method, but ambiguous values were obtained. This occurrence could be ascribed to the diversity of bacteria studied, which may secret various siderophores with differing iron affinities, and also to the highly alkaline conditions in which the maximum complexation capacity test was carried out. Ferreira et al. [17] argued that pH 9 could be used to test the maximum complexation capacity in basicity ranges typical of alkaline soils; however, it would be advisable to carry out a study of the stability of the siderophore–Fe complexation at different pH values. The pH will define the type of siderophore–Fe bonding. Hydroxamate functional groups bind to Fe^3+^ through the loss of a proton, which is conditioned by the pH of the medium [40]. Catecholate functional groups bind Fe^3+^ after the loss of two protons in neutral-alkaline pH via phenolic oxygens [41]. The pH could also affect the stability of the siderophore, and even competition for binding different metals, as siderophores such as DFOB are more likely to bind to Co^2+^ than Fe^3+^ at alkaline pH [42]. Therefore, pH is a limiting parameter when analyzing the maximum complexation capacity; the state of the functional groups and the stability of the compound at the pH of the study must be considered. The study of maximum Fe complexation capacity in alkaline soils should be studied at pHs where Fe chlorosis problems may occur. Iron precipitates as (hydr)oxides in soils at pH 7.5 in the presence of CaCO_3_ [24]. It would, therefore, be interesting to carry out the study starting from this pH, and not directly from pH 9.

Due to the results obtained in the maximum complexation capacity assay (Figure 3/Table 2), the bacterial strain RMC4 was chosen to perform an assessment and calculate more accurately the maximum Fe complexation capacity. The titration yielded a maximum complexation capacity of molar pyoverdine:Fe ratio of 1:1. A priori, these results may seem to contradict the results obtained previously; however, there are several factors to be considered. The titration was performed at 480 nm to observe the absorbance of the hydroxamate–Fe bond. This indicated that this group has a 1:1 iron complexing capacity; however, the rest of the possible complexing functional groups that may be present in the peptide chain of pyoverdine were not being observed. Furthermore, in Figure 4, which shows the formation of the complex, the increase in absorbance does not have a linear trend; two trend lines can be observed in the gradual increase in absorbance with different slopes, which could indicate the formation of different pyoverdine–Fe complexes where, probably, different functional groups would be involved. Also, once the maximum complexation point was reached, a decrease in absorbance was observed, which could be indicative of the degradation of the pyoverdine–Fe complex or the formation of another, more-stable pyoverdine–Fe complex not visible at the selected wavelength. Finally, it was shown that the selected strain is able to biosynthesize hemophore groups (Table 3), which indicates that pyoverdine was probably not the only complexing agent present in the medium. If the hypotheses presented were confirmed, we could potentially develop a promising iron biofertilizer with multiple complexing groups. Some of these groups might form more-stable iron complexes, while others could form less-stable ones. Consequently, when this iron biofertilizer is applied to plants, it could release the iron complexed in the less-stable groups more rapidly compared to the iron complexed in the more-stable groups. This might result in a slow release of iron, ensuring a continuous supply of iron to the plants even after the faster-release iron has been utilized.

The sequencing of the RMC4 genome revealed that the bacterium sequenced belonged to the species *P. monsensis*. The genome of *P. monsensis* RMC4 confirmed that this strain had potential as a plant growth-promoting rhizobacterium (PGPR) because it contains several beneficial genes for plants (Table 3). In fact, it is known that *P. fluorescens* strains are involved in plant growth-promoting activity by several mechanisms, for example, the production of siderophores and nutrient solubilization and mobilization, or in biological control through the production of antibiotics and fungicides [43,44,45]. Regarding the biocontrol potential of these rhizobacteria, Sehrawat et al. [46] reported on the beneficial effect of using antagonistic microorganisms (e.g., HCN producers) against pathogens, indicating they can be employed as a sustainable strategy, thus avoiding the use of pesticides. Type six secretion systems of pseudomonads have been proposed as a biocontrol trait against phytopathogenic bacteria [47]. The gene cluster for biosynthesis of the antifungal lokisin and the production of hydrocyanic acid identified in the genome of the RMC4 strain could give the bacteria the capacity for biological control. These results have also been previously reported in the genus *Pseudomonas* [48,49,50]. In addition, this strain has been shown to have insecticidal activity, which has also been observed in *P. fluorescens* towards agricultural pests [51]. To promote plant growth, microorganisms have also developed several mechanisms to mobilize and mineralize nutrients, such as iron or phosphate, that are not available for plant uptake [52,53]. In this sense, soil bacteria are known to produce small organic molecules, siderophores, under iron-limiting conditions through high-affinity interactions [54,55]. Our results indicate that *P. monsensis* RMC4 is involved in the biosynthesis of several iron siderophores, as previously found by Gu et al. [56] in the *P. koreensis* group. In addition, Fernandez et al. [57] informed that pseudomonads also could mobilize phosphate in the soil, and this was indeed observed in the RMC4 strain. Furthermore, phytohormones such as IAA and PAA, through catabolism, play an important role in plant growth and development and are involved in the interactions between microorganisms and plant roots [58,59,60]. The presence in the *P. monsensis* RMC4 strain of both IAA biosynthesis and PAA degradation pathways could be important for the PGPR activity. Therefore, the present study provides a more comprehensive view of the capacity of the *P. monsensis* RMC4 strain as a plant growth-promoting biofertilizer and its potential as a biostimulant.

The results described above showing the highest production of siderophores and pyoverdine and higher iron complexation capacity show that *Pseudomonas monsensis* RMC4 is an excellent candidate to be used as an Fe biofertilizer and able to mobilize iron. Furthermore, the finding that the genome encodes many other plant growth-promoting traits highlights its use as a polyvalent agricultural inoculant. A kinetic study was performed (144 h) where some carboxylic acids with biostimulant properties [22] were measured (glutamic acid, acetic acid, aminobutyric acid, IAA, and succinic acid); data are shown in Figure 5. The limiting factor in the production of the different acids was the Fe deficiency of the bacteria, which affected many metabolic processes of the bacteria, such as protein and nucleic acid synthesis. Glutamic acid is an amino acid with biostimulant properties, possibly related to the fact that it is the central product in the nitrogen metabolism pathway [61], is involved in chlorophyll biosynthesis [62], and has been shown to exert a positive effect on Fe uptake in tomato plants with lime-induced Fe deficiency [63]. As shown in Figure 5C, the initial concentration of glutamic acid decreased until 48 h and then increased in a linear progression until the end of the experiment. Glutamic acid is known to be involved in the biosynthesis of pyoverdine, being a component of the side chain, and could be modified to succinimide, catalyzed by pyoverdine I decarboxylase (PvdN), or to α-ketoglutarate, catalyzed by pyoverdine aminotransferase (PtaA) [64]. The concentration of pyoverdine increased until 48 h, then remained stable until the end of the experiment (Figure 5F). As mentioned above, the biosynthesis of pyoverdine requires a very high energy expenditure for the organism, probably after 48 h, and not having obtained Fe, it is likely that the bacteria are no longer producing pyoverdine, keeping its concentration stable and possible causing metabolites used for its biosynthesis (such as glutamic acid) to increase in concentration. Acetic acid can be produced in the metabolic pathway of the bacteria. High concentrations of acetic acid are an important physiological stress factor in cells [65]. Acetic acid had a maximum at 12 h (Figure 5A), then remained stable with values around 2 mmol·L^−1^, and after 72 h, its values increased to a maximum of 4 mmol·L^−1^. The initial values were probably due to natural generation of the bacteria´s metabolism, and the maximum value in the final time could be indicative of physiological damage caused by severe Fe deficiency and failure of Fe acquisition strategies to work. Gamma-aminobutyric acid (GABA) is a phytohormone secreted by plants with abiotic stress regulation functions [66]. Several studies have reported its positive effects in horticultural crops under abiotic stresses; in melon plants with saline-alkaline stress, it induced increased growth, reduced oxidative stress levels, and increased antioxidant enzymes [67]. In bacteria, GABA is synthesized from the α-decarboxylation of L-glutamic acid. The GABA concentration value was around 2 mmol·L^−1^ until 26 h, then the concentration decreased until it was not detected after 52 h. The variation in values was similar to that observed for glutamic acid, under Fe deficiency conditions; the normal functioning of metabolism was disrupted, leading to the inability to metabolize GABA. Indol-3-acetic acid is a well-known phytohormone involved in the regulation of growth, stem elongation, and seed germination, among other functions. In bacteria, IAA synthesis plays a key role in plant–microorganism interaction. These interactions could promote plant phytostimulation but also could be pathogenic [56]. In the present experiment, high concentrations of IAA were detected at 26 h, then ceased to be produced after 34 h. It was demonstrated that the RMC4 bacterium was able to produce IAA, a compound with PGP characteristics that could have a biostimulant effect on the plant. This result agrees with that obtained for the PGPR characterization (Table 3): the bacterium possessed the *iaaHM* gene, whose function is the biosynthesis of auxins (such as IAA). Succinic acid was the carbon source used by the bacteria in this experiment. The concentration of this compound decreased until it was not detected after 30 h (Figure 5E). Perhaps, as it was the only carbon source available to the bacteria, this compound was consumed by the bacteria and once completely consumed, the bacterial metabolism was deregulated, causing the chain reaction that was observed with the other compounds (it stopped producing GABA, IAA, and pyoverdine). Regarding pyoverdine, initially, its production was slow. After 12 h, the pyoverdine concentration was less than 10 µmol·L^−1^. Probably due to the activation of the Fe acquisition mechanism, the production of pyoverdine increased substantially until 48 h, when it reached a concentration of 85 µmol·L^−1^, which means that in the first 12 h, the production of 0.83 µmol·L^−1^·h^−1^ was observed, and in the following 36 h, the pyoverdine production rate was 2.08 µmol·L^−1^·h^−1^, an increase of 2.5 fold in the production rate. Possibly due to the deregulation of the bacteria´s metabolism (as explained before), from 48 h until the end of the experiment (144 h), pyoverdine production remained stable, obtaining a concentration of 108 µmol·L^−1^ at the end of the experiment. A further 23 µmol·L^−1^ was obtained from 48 h to 144 h, resulting in a production rate of 0.24 µmol·L^−1^·h^−1^ of pyoverdine in the last part of the experiment. With these results, three phases of pyoverdine production by the bacterium were observed. The first phase may correspond to metabolic activation; the bacterium was aware of the need for Fe and activated the strategies for acquiring this element. The second phase corresponds to the metabolic zenith; the production of pyoverdine is the fastest, as a result of the activation of Fe acquisition strategies. Finally, the last phase corresponds to metabolic collapse, since the Fe acquisition strategies have not worked and the bacterial metabolism has collapsed, which will probably lead to the death of the bacterium. For the biotechnology industry interested in optimizing pyoverdine production, the challenge to overcome is to optimize the parameters that affect pyoverdine production (temperature conditions, carbon source, nitrogen source, iron concentration, pH, etc.) in order to extend the metabolic zenith phase as much as possible and try to increase the production per hour in this phase.

## 4. Materials and Methods

### 4.1. Bacterial Isolation

Bacteria were isolated from the rhizosphere of different horticultural plant species: pepper (*Capsicum annuum*), tomato (*Solanum lycopersicum*), and pumpkin (*Curcubita* sp.). Roots were weighed and immersed in saline solution (8.5% NaCl). After vigorously shaking and serial decimal dilutions, the supernatants were used to inoculate plates of Sucrose-Asparagine (SA) culture medium, a medium that is selective for pseudomonads and stimulates pyoverdine production [68]. Ampilicin (100 µg·mL^−1^) and Cycloheximide (100 µg·mL^−1^) were added to the medium to increase selectivity and to avoid the growth of eukaryotes. After two days of incubation, colonies showing a yellow/green fluorescence were selected as potential *Pseudomonas* isolates. Colonies that did not show fluorescent pigment were discarded. Those that survived temperatures of 37 °C were discarded to avoid potential pathogens. In addition, using the Box-PCR technique as described by Gutiérrez-Barranquero et al. [69], sibling strains were discarded. Those bacteria showing higher siderophore production than the control bacterium *Pseudomonas fluorescens* F113 [28] according to the halo formation in the CAS agar assay [29] were selected as potential biofertilizer candidates. Strains were identified as *Pseudomonas* spp. by amplification and sequencing of the 16S RNA gene.

### 4.2. Culture Conditions and Siderophore and Pyoverdine Production

The selected bacterial strains were grown in 10% phosphorus minimal medium succinate (MMS), described as optimized for siderophore production by bacteria, with the following composition (reagents obtained from Panreac (Barcelona, Spain) except where indicated): 0.6 g·L^−1^ dipotassium hydrogenphosphate trihydrate; 0.3 g·L^−1^ monopotassium phosphate; 0.2 g·L^−1^ magnesium sulfate heptahydrate; 1.0 g·L^−1^ ammonium sulfate; and 4.0 g·L^−1^ succinic acid (Sigma-Aldrich (St. Louis, MO, USA)), pH fixed at 7.00 ± 0.01 [19,31]. Culture conditions were as described by Vindeirinho et al. [31]. In brief, starter cultures were obtained by inoculating 2 loops of culture in MMS with ~3.7 µmol·L^−1^ FeCl_3_ ∙ 6 H_2_O (Panreac), at 30 °C, 150 rpm, for ~8 h. Pre-cultures were prepared also in MMS with Fe 0.37 µmol·L^−1^, by inoculating an appropriate volume of the starter culture, and incubated under the same conditions described above, for ~16 h until the bacteria reached exponential phase (optical density OD_600_ ~ 1.0). Finally, cultures designated for siderophore production were prepared in 200 mL MMS without Fe, by inoculating an appropriate volume of the pre-culture until an initial OD_600_ of ~ 0.1. Bacteria were incubated for ~48 h under the same conditions described above. Subsequently, samples were centrifuged (3000× *g*, 15 min, at 25 °C), filtered using a 0.45 µm cellulose nitrate membrane filter (Labbox Labware S.L. Barcelona, Spain) and stored protected from light at −20 °C until siderophore quantification. The quantification of siderophores was conducted using the CAS liquid assay method originally outlined by Schwyn and Neilands [29], as amended by Mehnert et al. [30]. In brief, 1.5 mL of 1 mol·L^−1^ FeCl_3_ ∙ 6 H_2_O dissolved in 10 mol·L^−1^ HCl (Merck, suprapur) was mixed with 7.5 mL of 2 mol·L^−1^ CAS solution; then, the mixture was added slowly to 6 mL of 10 mol·L^−1^ cetyltrimethylammonium bromide (purchased from Sigma-Aldrich). Simultaneously, 9.76 g 2-(N-morpholino)ethanesulfonic acid (MES) (Sigma-Aldrich) was dissolved in 80 mL water, and pH was adjusted to 5.6 with 50% KOH. Water was added to attain a final volume of 85 mL, and this MES buffer solution was then combined with the dye solution. After 4 h, 150 µL of the culture supernatant and 150 µL of metal CAS solution were mixed, and OD was measured at 630 nm. To quantify siderophores, a commercial siderophore known as DFOB (Sigma-Aldrich) was utilized as standard, and the concentrations within culture supernatants were expressed in “DFOB-equivalents”. A standard curve was constructed by plotting the discolorization (*d*) of the metal CAS solution at 630 nm as a function of siderophore concentration (Equation (1)). Sterile culture medium was used as reference solution (*A_ref_*), and zero absorbance (*A*_0_) was performed using a mixture of the metal CAS solution and 2 mol·L^−1^ DFOB. Presuming an association/dissociation equilibrium, data were fitted using (Equation (2)) with *y_max_* set at 100. Both the methodology and the equations were obtained from the protocol described by Mehnert et al. [30].

(1) $$d=\frac{A_{ref}-A_{supernatant}}{A_{ref}\;–\;A_{0}}$$

(2) $$y=y_{max}\cdot \left(1-e^{-kx}\right)$$

The concentration of pyoverdine was measured by UV-VIS spectroscopy. The chromophore group, common in the pyoverdines produced by *Pseudomonas* genera, has a maximum absorption peak between 380–400 nm; thus, the concentration was determined using the Lambert–Beer law, using the molar extinction coefficient (ε) of 16,000 L·mol^−1^ ∙ cm^−1^ [19].

To avoid Fe contamination, all glassware was soaked with HCl (VWR, Normapur) 6M overnight and, afterwards, washed with ultrapure water (Milli-Q system, Bedford, MA, USA).

### 4.3. Iron Complexation Capacity Assays

The complexation capacity assay was performed as described by Villén et al. [70]. To a fixed volume of the supernatant, increasing concentrations (from 0 to 16 mg·L^−1^) of FeCl_3_ ∙ 6 H_2_O were added, then the pH was adjusted to 9.00 ± 0.01. The solution was allowed to stand for 3 h, then the pH was adjusted again to 9. Subsequently, the solution was left in the dark for 24 h. Afterwards, pH was adjusted once more. Then, the mixture was centrifuged (10,000× *g*, 15 min) and filtered by 0.45 µm pore-size nylon membrane. Finally, the final volume was adjusted to 50 mL, and the concentration of Fe was determined by atomic absorption spectroscopy with flame atomization (Perkin-Elmer Analyst 800; Shelton, CT, USA).

### 4.4. Titration of Bacterial Secretion

The assessment protocol described by Yunta et al. [71] was followed with modifications. Pyoverdine was obtained as described in Section 4.2. The pH was adjusted to 8, and the concentration of pyoverdine was measured as described above. The experimental solution (10 mL) was placed in a 50 mL thermostatic (25.0 ± 0.5 °C) jacketed reaction vessel provided with an airtight cap fitted with a gas inlet and outlet tubes, combined pH glass electrode, a spectrode, two piston burets (tips placed below the surface of the solution), and a magnetic stirrer. The photometric titration consisted of the gradual addition of Fe^3+^ standard solution to the pyoverdine until the absorbance at 480 nm remained constant. Potentiometric measurements were performed with Metrohm 719 and 721 potentiometers (precision of 0.1 mV) combined with a pH glass electrode that kept the pH constant at 8.0, while a NaOH 0.2 mol·L^−1^ solution was automatically added if necessary. Photometric titrations were carried out using a Metrohm 662 photometer (resolution of 10 ± 0.1 nm) with a light spectrode of path length 2 × 10 nm. Both potentiometers were controlled by the software for PC Tiamo 2.5 (Metrohm AG, Switzerland). The titration was performed in triplicate.

### 4.5. Evaluation of Temporal Variation in Organic Acid Concentrations Secreted by Bacteria

A kinetic assay was carried out to evaluate temporal changes in organic acid concentrations secreted by the selected bacteria. The analysis was focused on the identification of 11 carboxylic acids, including monocarboxylic acids (MCAs) and polycarboxylic acids (PCAs): acetic, lactic, oxalic, citric, aminobutyric, succinic, malic, gluconic, fumaric, pyruvic acids, and IAA. For this, the experiment was carried out for 144 h (6 days), and samples were taken every 4 h. Zero time corresponded to the cultures to produce siderophores, after performing the initial pre-culture and culture procedures described in Section 4.2. For its evaluation, a previous sample treatment was necessary. Briefly, 1.0 mL of sample was loaded onto a Strata-X-AW 33 µm polymeric weak anion cartridge (SPE) (Phenomenex, Torrance, USA), previously conditioned with 5 mL of methanol (MeOH) (Sigma-Aldrich) and 5 mL of 25 mM tris(hydroxymethyl)aminomethane-acetate (Tris-OAc) (Sigma-Aldrich) (pH = 7.5) for MCAs and 25 mM MES (Sigma-Aldrich) (pH = 4.5) for PCAs at about 1 mL·min^−1^ by means of a suction system. The SPE cartridge was then washed with 5 mL of a mixture of 25 mM Tris-OAc:MeOH (90:10, *v*/*v*) for MCAs and 25 mM Tris-OAc:MeOH (90:10, *v*/*v*) in the case of PCAs. The rinse was discarded, and after 10 min of drying time, the analytes were eluted with 2 mL of 25 mM Tris-OAc: 0.01M HCl (10:90, *v*/*v*) mixture for MCAs and 0.1 M HCl for PCAs. The resulting solution was passed through a nylon 0.45 µm syringe filter, and 10 µL was injected into the chromatographic system. Analysis was achieved by high-performance liquid chromatography coupled to a refractive index detector (HPLC-RID) system (1260 Infinity model Agilent Technologies, Waldbronn, Germany). A Bio-Rad Aminex HPX-87 H column (300 × 7.8 mm, 9 µm) was used, protected by a guard column from Phenomenex. Analysis conditions were set as follows: the mobile phase was sulfuric acid 5 mM, the flow rate was 0.5 mL·min^−1^_,_ the column temperature was set at 60 °C, and the temperature of the refractive index detector was at 50 °C in positive polarity mode.

During sampling times, the concentration of pyoverdine was also measured by UV-Vis spectrophotometry, as explained in Section 4.2. The experiment was conducted in triplicate.

### 4.6. Sequencing and Analysis of the Genome of Strain RMC4

The genomic DNA of strain RMC4 was extracted using the NucleoSpig^®^ Microbial DNA kit (Macherey-Nagel, Düren. Germany), and the quality was determined on an agarose gel and quantified using a Qubit fluorimeter (Invitrogen, Carlsbad, CA, USA). The genome was analyzed on a MinION sequencer (Oxford Nanopore Technologies, UK). The library was prepared with 1 μg of DNA and the Nanopore Ligation Sequencing kit (SQK-LSK-110). The library was loaded onto a MinION flow cell (R9.4.1 pores) following the manufacturer’s recommendations. Subsequently, sequencing was performed via MinKNOW software (v22.03.6). Guppy (v6.0.7) was used to carry out the base calling step, which consists of translating raw signal data into nucleotide sequences in FASTQ format, and reads shorter than 5000 nts were excluded before further analysis [72]. For bioinformatic analysis, NanoPack [73] was applied to evaluate the read length and ASE calling quality. Additionally, the ONT reads were filtered, and the adapter sequences were removed with Porechop (v0.2.4). The novo assembly was performed using Flye (v2.9, [74]), and SquezzeMeta was used to annotate the genome and determine the taxonomic classification [75]. In addition, the Type-Strain Genome Server (TYGS) platform [76] was used to determine whether the genome sequenced corresponded or not to a known bacterial species, through the percentage of DNA–DNA hybridization (%DDH). The *P. monsensis* RMC4 genome has been submitted to the NCBI database and is available under BioProiect accession number PRJNA1028413. Secondary metabolite biosynthesis clusters were identified using the AntiSMASH (v7.0) web application [77]. The genome was also searched using subsystems technology (RAST server, [78]) in order to identify genes and gene clusters implicated in plant growth promotion.

### 4.7. Statistical Analysis

IBM SPSS Statistics 24.0 software (SPSS Inc., Chicago, IL, USA) was used for one-way analysis of variance (ANOVA). Siderophore and pyoverdine production were compared using Duncan’s test for *p* < 0.05.

## 5. Conclusions

In the present work, RMC4 was selected from a large group of bacteria isolated from horticultural soils according to their siderophore production, corresponding mainly to pyoverdine. The Fe chelating capacity was evaluated at alkaline pH, elucidating that the bacterial secretion had the capacity to form an Fe complex in a 1:2 molar ratio (secretion:Fe), which was explained not only by the pyoverdine but also by the presence of other siderophores of compounds able to complex Fe in the studied conditions. In addition, RMC4 showed plant biostimulant characteristics according to its high production of IAA or glutamic acid and the gene clusters related to phosphorus mobilization/solubilization and the production of antibiotics and antifungals detected by genomic analysis. These results contribute to the existing knowledge of *Pseudonomas* as a siderophore-producing bacterium and bring novel insights to the identification of eco-friendly alternatives to synthetic ligands for Fe chelation and their potential use to alleviate Fe chlorosis in crops.

Future experiments involving plants can contribute to advancing our comprehension of the effectiveness of Fe complexed by RMC4 secretions (mainly pyoverdine–Fe) as a biofertilizer in calcareous environments and assessing the biostimulant impact of RMC4 on plant growth.

## Figures and Tables

**Table 1:** Measure of halo formation (mm) in the CAS agar assay. Strain F113 was used as a control bacterium.

Strains	Halo Formation (mm)	Strains	Halo Formation (mm)
F113	10		
HFL1	15	RMT4	
HFL3	30	RMT6	13
HFL4	10	RMT7	5
RMC2	10	RMT9	15
RMC4	25	RMT12	20
RMC5	12	RMP5	10
RMC6	7	RMP9	12
RMC8	8	RKP1	7
RMC9	23	RKP2	17
RMT2	10	RKP3	20

**Table 2:** Maximum number of Fe atoms that can be complexed by each bacterial cell–free supernatant.

Strain	Soluble Fe:Fe Added
F113	1:1
RMT9	1:2
RKP1	1:1
RKP2	1:2
RKP3	3:4
RMC4	2:1
RMC9	2:3
HFL3	1:3

**Table 3:** Identification of the PGPR characteristics of RMC4 strain.

Possible Specie	Genes/Clusters	Function	PGP Category
*Pseudomonas monsensis*	*Cluster 1*	Type NRPS/lokisin (78%)	Antifungal
	*fecAR*	Transport of iron dicitrate (III)	Iron siderophore receptor protein
	*fitD*	Insect toxin	Toxin
	*hasDEF*	Hemophore biosynthesis	Siderophores
	*hcnABC*	Hydrocyanic acid biosynthesis	Biocontrol
	*hcp (T6SS)*	Type VI secretion system	Biocontrol
	*iaaHM*	Auxin biosynthesis	Phytohormone modulation
	*paaFIKY*	Phenylacetic acid degradation	Interaction with the environment
	*phnBCDENWXZ*	Phosphate transport	Nutrient mobilization (P)
	*phoBDHH2LPQRU*	Phosphate transport	Nutrient mobilization (P)
	*pqqABCDE*	Pyrroloquinoline quinone biosynthesis	Nutrient solubilization (P)
	*pstABCS*	Phosphate transport	Nutrient mobilization (P)
	*pvdE*	Pyoverdine	Nutrient mobilization (Fe)
	*ubiA*	Production of 4-hydroxybenzoate	Antibiotic

## Data Availability

The data presented in this study are openly accessible at e-cienciaDatos, https://edatos.consorciomadrono.es/.
